# Broad-Spectrum Small-Molecule Inhibitors of the SARS-CoV-2 Spike—ACE2 Protein–Protein Interaction from a Chemical Space of Privileged Protein Binders

**DOI:** 10.3390/ph15091084

**Published:** 2022-08-30

**Authors:** Sung-Ting Chuang, Peter Buchwald

**Affiliations:** 1 Diabetes Research Institute, Miller School of Medicine, University of Miami, Miami, FL 33136, USA; 2 Department of Molecular and Cellular Pharmacology, Miller School of Medicine, University of Miami, Miami, FL 33136, USA

**Keywords:** antiviral, coronavirus, COVID-19, delta (B.1.617.2) variant, HCoV-NL63, omicron (B.1.1.529) variant, protein–protein interaction, SARS-CoV-2, spike protein, variants of concern

## Abstract

Therapeutically useful small-molecule inhibitors (SMIs) of protein–protein interactions (PPIs) initiating the cell attachment and entry of viruses could provide novel alternative antivirals that act via mechanisms similar to that of neutralizing antibodies but retain the advantages of small-molecule drugs such as oral bioavailability and low likelihood of immunogenicity. From screening our library, which is focused around the chemical space of organic dyes to provide good protein binders, we have identified several promising SMIs of the SARS-CoV-2 spike—ACE2 interaction, which is needed for the attachment and cell entry of this coronavirus behind the COVID-19 pandemic. They included organic dyes, such as Congo red, direct violet 1, and Evans blue, which seem to be promiscuous PPI inhibitors, as well as novel drug-like compounds (e.g., DRI-C23041). Here, we show that in addition to the original SARS-CoV-2 strain, these SMIs also inhibit this PPI for variants of concern including delta (B.1.617.2) and omicron (B.1.1.529) as well as HCoV-NL63 with low- or even sub-micromolar activity. They also concentration-dependently inhibited SARS-CoV-2-S expressing pseudovirus entry into hACE2-expressing cells with low micromolar activity (IC_50_ < 10 μM) both for the original strain and the delta variant. DRI-C23041 showed good therapeutic (selectivity) index, i.e., separation between activity and cytotoxicity (TI > 100). Specificities and activities require further optimization; nevertheless, these results provide a promising starting point toward novel broad-spectrum small-molecule antivirals that act via blocking the interaction between the spike proteins of coronaviruses and their ACE2 receptor initiating cellular entry.

## 1. Introduction

COVID-19, the coronavirus disease caused by the severe acute respiratory syndrome coronavirus 2 (SARS-CoV-2), is an ongoing pandemic that continues to spread worldwide [1,2]. As of mid-2022, there have been over 500 million confirmed cases and 6 million death cases reported by the World Health Organization (WHO). Since a gold-standard treatment is still lacking, it is urgent that new antiviral drugs are developed, especially oral ones that would allow more widespread use, including by those who are either not willing to be vaccinated or are unable to do so (e.g., due to some pre-existing medical condition). The sites of viral attachment and entry are of particular interest as possible therapeutic targets [3] since they are the first steps of the replication cycle and occur at a relatively accessible extracellular site [4]. SARS-CoV-2 uses the human angiotensin converting enzyme 2 (hACE2) as cell entry receptor, attaching itself via the receptor-binding domain (RBD) of its spike (S) protein [5,6,7,8]. SARS-CoV-2 RBD seems to have a higher ACE2-binding affinity than SARS-CoV(-1) due to some residue changes in the RBD stabilizing two virus-binding hotspots at the RBD–ACE2 interface [9]. The SARS-CoV-2 spike is a highly glycosylated homotrimer. It is post-translationally cleaved into S1 and S2 subunits, with S1 consisting of the amino-terminal domain and the RBD and S2 including the trimeric core and being responsible for membrane fusion [10,11]. The RBD switches between a stand-up position needed for binding to the receptor and a lie-down position used for evading the immune attack [5,12]. Since attachment of the spike (S) protein to its ACE2 receptor is a key step of viral infection, agents that block this, such as neutralizing antibodies or viral entry inhibitors, can be used to prevent infection [13,14,15].

Although vaccination has been successfully shown to control the outbreak of COVID-19, viral variants of increased fitness that facilitate the virus spread and transmission rate [16] and show resistance to the immunity induced by current vaccines against COVID-19 have already arisen [17,18]. To date, five such variants have been defined as variants of concern (VoC) due to their increased transmissibility, higher disease severity, and resistance to neutralizing antibodies, including those elicited by the existing clinically approved vaccines [19,20,21,22,23]. In chronological order, they are: *alpha* (B.1.1.7), *beta* (B.1.351), *gamma* (P.1), *delta* (B.1.617.2), and *omicron* (B.1.1.529). The delta variant, which replaced alpha to become the predominant lineage around October 2020, is characterized by spike protein mutations T19R, Δ157–158, L452R, T478K, D614G, P681R, and D950N. These amino acid mutations play a crucial role in cell infection and may affect the immune responses directed against the main antigenic regions of receptor-binding proteins (452 and 478) and the deletion part of the N-terminal domain (157–158) [2]. The delta variant exhibited reduced sensitivity to antibodies versus the alpha variant [24]. After a single dose application, the Pfizer/BioNTech (BNT162b2) and AstraZeneca (ChAdOx1 nCoV-19) vaccines were less effective in people infected with the delta variant than in those infected with the alpha variant [25]. In November 2021, the omicron variant was identified; it had an alarmingly high number of mutations (>30) in its spike protein, including >15 in its RBD, which is the principal target of neutralizing antibodies, and it continues to spread around the world [26]. Because of the large number of spike mutations, the effectiveness of current COVID-19 vaccines and antibody therapies is likely compromised [27]. SARS-CoV-2 clearly has the potential to continue developing and become increasingly more infectious and less responsive to available therapies.

Development of effective broad-spectrum oral medications could have significant bearing on the coronavirus pandemic as such treatments could be started easily as soon as the first symptoms manifest. Remdesivir was the first small-molecule antiviral approved by the United States Food and Drug Administration (FDA) for the treatment of COVID-19 in October 2020, but it must be given intravenously [28]. Several attempts at repurposing (repositioning) approved drugs as possible small-molecule antiviral agents for SARS-CoV-2 have been pursued in the past two years; however, only very limited success has been achieved so far. For example, WHO’s large Solidarity trial found that repositioned antiviral drugs—including hydroxychloroquine, lopinavir, remdesivir, and interferon-β1—had little or no effects in patients hospitalized with COVID-19 [29]. Thus, there is an ongoing need to develop novel drugs that can combat such infections, including those that might arise in the future [30]. Molnupiravir and nirmatrelvir, two small-molecule drugs that exert antiviral effects via inhibition of viral reproduction and protease activity, respectively, have received emergency use authorizations (EUAs) from the FDA in late 2021 as COVID-19 treatments. Molnupiravir is an oral prodrug of N4-hydroxycytidine, a ribonucleoside that exerts its antiviral action by introducing copying errors during viral RNA replication. Nirmatrelvir is an oral peptidomimetic inhibitor of M^pro^ (the main SARS-CoV-2 protease) [31] that binds directly to the SARS-CoV-2 M^pro^ active site [32] and is approved in combination with ritonavir being sold as Paxlovid. 

In light of our past work on small-molecule inhibitors of protein–protein interactions (SMIs of PPIs) [33,34,35], we focused on SMIs of the CoV spike—hACE2 PPI as possible viral entry inhibitor antivirals [36,37,38]. Small molecules traditionally were not considered to be likely PPI modulators because protein surfaces tend to lack binding pockets that could make adequate binding possible for them. This changed during the past two decades as an increasing amount of evidence confirmed that SMIs can be effective against at least certain PPIs. SMIs which are or have been in preclinical development target more than 40 PPIs [39,40,41], and three such SMIs (venetoclax [42], lifitegrast [43], and fostemsavir [44]) were recently approved by the FDA for clinical use. Notably, enfuvirtide, maraviroc, and fostemsavir, which are all PPI inhibitors, are approved for clinical treatment against HIV-1, showing that such strategies can be successful for antiviral drug discovery. In particular, fostemsavir, which acts via blocking the gp120–CD4 interaction [44] and was approved for clinical use as an antiretroviral by the FDA in 2020 (Rukobia), provides strong support for the feasibility of the PPI SMI antiviral concept [15]. Compared to antibodies, SMIs could become antiviral therapies that are more patient-friendly (as they are more suitable for oral or inhaled formulations), less immunogenic (as they are not foreign proteins introduced into the body), more controllable (as they have shorter half-lives and better biodistributions), and possibly even more broadly active (i.e., less strain- and mutation-sensitive) [15]. Oral drug delivery is the most convenient, cost effective, and safe route of administration [45], ensuring higher patient compliance and therefore being most suitable for broadly acceptable and long-term preventive use [46,47,48], which is also important in the control of viral diseases. SMIs could also be directly delivered into the respiratory system via inhaled or intranasal administration to obtain higher local concentration, which cannot be done with antibodies and could be relevant for COVID-19 treatments. Moreover, SMIs could be broadly active with multi-strain or even pan-CoV inhibitory activity, which is less likely with antibodies that tend to be highly specific [49,50].

As part of our immunopharmacology work, we started a search for SMIs of co-signaling PPIs as potential immunomodulatory agents, and we focused on the chemical space of organic dyes because they tend to have good affinity for proteins [51] and contain structural elements that are considered privileged structures for protein binding [52,53,54]. We hypothesized that this is a better starting point to identify SMIs of PPIs than most commonly available drug-like libraries, and indeed, we found several organic dyes and novel drug-like small-molecule compounds derived from them, designated as DRI-C series, that show promising inhibitory activity against immune checkpoint PPIs of interest such as CD40–CD40L [33,34,35,41]. More recently, following the outbreak of the COVID-19 pandemic, we have shown that some of these dyes and DRI-C compounds possess potential antiviral ability against the original SARS-CoV-2 strain via inhibiting the entry of the virus [36]. Interestingly, we also found that methylene blue, which is approved by the FDA for clinical use as treatment for methemoglobinemia, could be useful as a repurposed antiviral agent against COVID-19, including its delta variant [37,38]. In the present study, we further investigated the activity of some of our most promising drug-like novel compounds (DRI-C23041 and DRI-C24041) as possible inhibitors of CoV entry, including for VoCs such as delta and omicron, while also including some of the organic dyes that showed the most promising inhibitory activity for comparison.

## 2. Results

### 2.1. Inhibition of SARS-CoV-2 Spike—hACE2 PPI (Original Strain)

**Figure 1:**
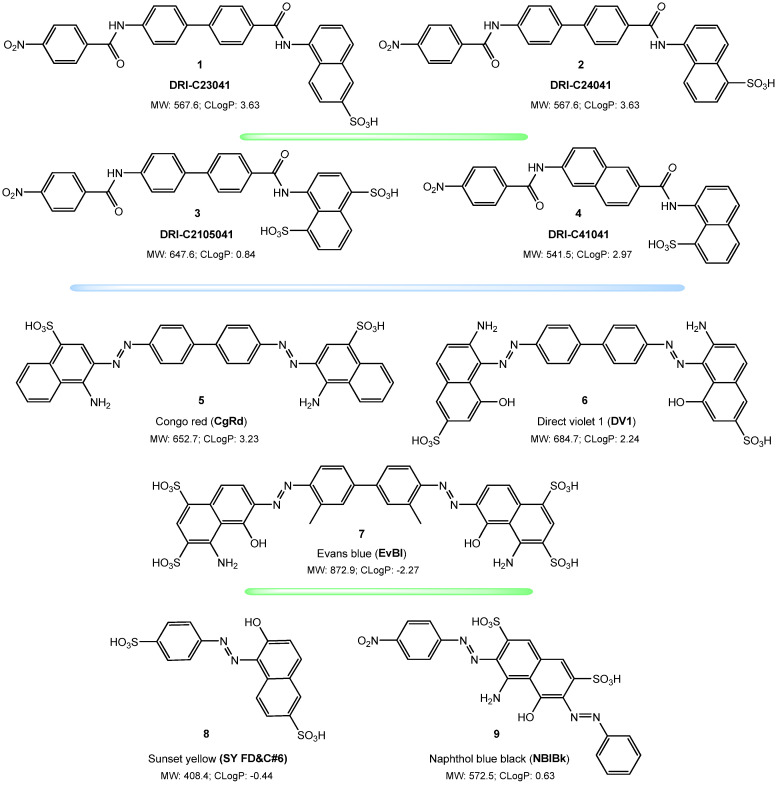
**Chemical structures of the compounds included in the present study.** Structures of the DRI-C compounds (**1**–**4**) and organic dyes (**5**–**9**) included in the present study are shown with molecular weight (MW) and ChemDraw calculated octanol-water partition coefficients (CLogP) included as size and hydrophobicity indicators.

**Figure 2:**
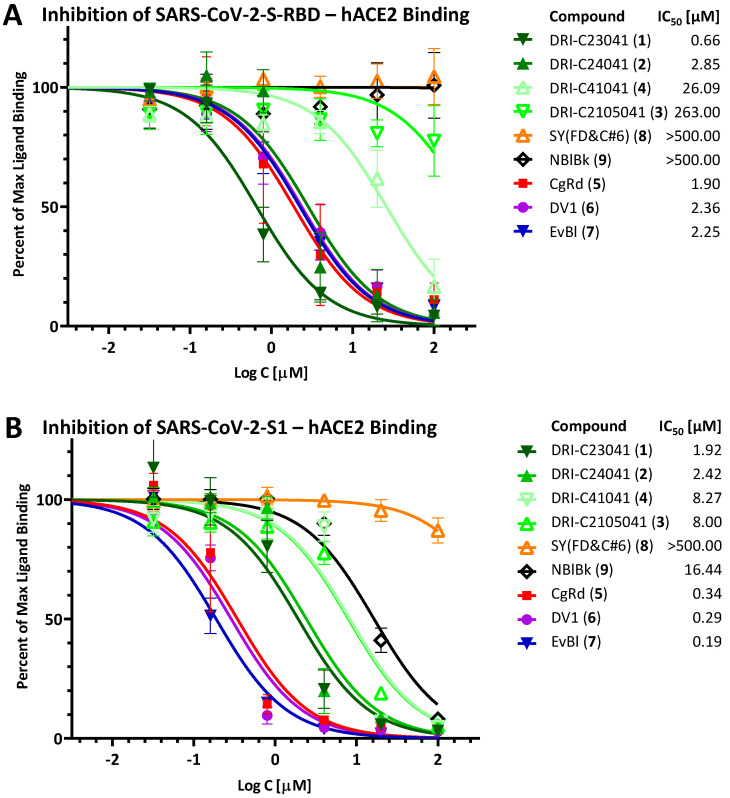
**Inhibition of SARS-CoV-2-S binding (original strain) to hACE2.** Concentration-response curves obtained for the inhibition of the PPI between SARS-CoV-2-S-RBD (**A**) or SARS-CoV-2-S1 (**B**) and hACE2 in cell-free ELISA-type assays by the compounds tested here (**1**–**9**). Data (symbols) are mean ± SD from three experiments in duplicates. IC_50_ values obtained from fitting with standard sigmoid curves are shown in the legend confirming that several of the DRI-C compounds and organic dyes included here showed promising low micromolar or even sub-micromolar activity (IC_50_ < 1 μM).

Following up on our previous work [36], we reconfirmed here that two of our DRI-C compounds, DRI-C23041 (**1**) and DRI-C24041 (**2**) (Figure 1) inhibit the interaction between the SARS-CoV-2 spike RBD and hACE2 in classic concentration-responsive manner for the original strain in our ELISA-based assay, with DRI-C23041 showing particularly promising (sub-micromolar) activity (IC_50_ = 0.66 μM), but DRI-C24041 also having low micromolar activity (IC_50_ = 2.85 μM) (Figure 2A). Thus, these compounds are indeed promising SMIs of the SARS-CoV-2-S—hACE2 PPI, which is essential for the attachment and entry of this coronavirus. We also reconfirmed the activity of three organic dyes identified earlier as having inhibitory activity and included here as comparators of possible interest: Congo red (**5**, CgRd), direct violet 1 (**6**, DV1), and Evans blue (**7**, EvBl) (IC_50_s of 1.90, 2.36, and 2.25 μM, respectively) (Figure 2A). Two other organic dyes, sunset yellow FCF (**8**, SY(FD&C#6)) and naphthol blue black (**9**, NBlBk), were included as negative controls and indeed showed no activity in this assay (IC_50_ > 500 μM). We also included two other DRI-C compounds of slightly different structure (Figure 1) to confirm our structure-activity relationship (SAR) assumptions, and they indeed showed strongly diminished inhibitory activity against this PPI involving the RBD of the original strain: DRI-C41041 (**4**) and DRI-C2105041 (**3**), with IC_50_ values of 26.09 and 263.00 μM, respectively.

Inhibitory activities were also assayed using not the RBD but the S1 fragment of the spike protein (Figure 2B). The activity of DRI-C23041 and DRI-C24041, as well as of the dyes CgRd, DV1, and EvBl, remained within the same low-micromolar range (0.2–3.0 μM; Figure 2B), and the negative control SY (FD&C#6) showed no inhibition (IC_50_ > 500 μM). However, DRI-C41041, DRI-C2105041, and NBlBk showed some activity in this assay with IC_50_ values in the mid-micromolar range (8–20 μM; Figure 2B). On one hand, this could be due to the somewhat weaker binding of S1 as used here to hACE2 than that of the RBD fragment (see Figure 2 in [36]). On the other hand, this may illustrate that slightly different structural requirements are needed to inhibit RBD and S1 binding—see also binding of the VoC PPIs below.

### 2.2. Inhibition of SARS-CoV-2 Spike—hACE2 PPI (Variants of Concern including Delta and Omicron)

**Figure 3:**
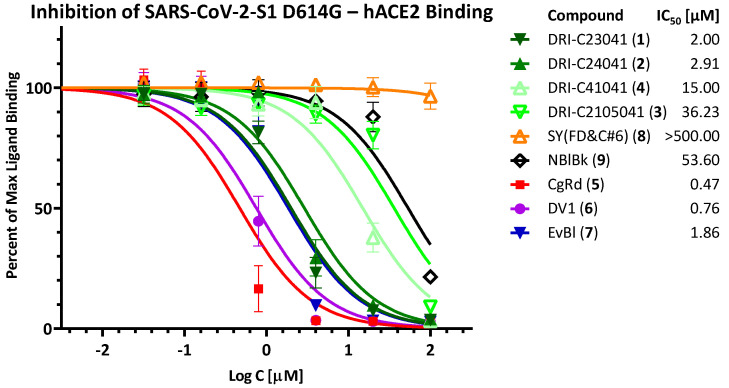
**Inhibition of SARS-CoV-2-S binding (D614G mutant) to hACE2.** Concentration-response curves obtained for the inhibition of the PPI between SARS-CoV-2-S1 (D614G) and hACE2 as done for the original strain in Figure 2.

**Figure 4:**
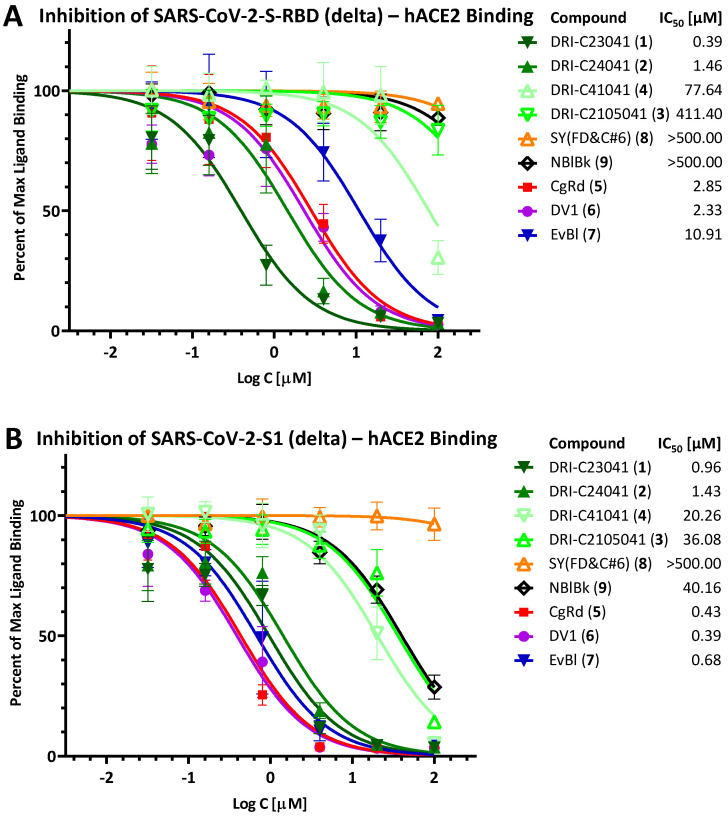
**Inhibition of SARS-CoV-2-S binding (delta, B.1.617.2) to hACE2.** Concentration-response curves obtained for the inhibition of the PPI between SARS-CoV-2-S-RBD (delta) (**A**) or SARS-CoV-2-S1 (delta) (**B**) and hACE2 as done for the original strain in Figure 2.

**Figure 5:**
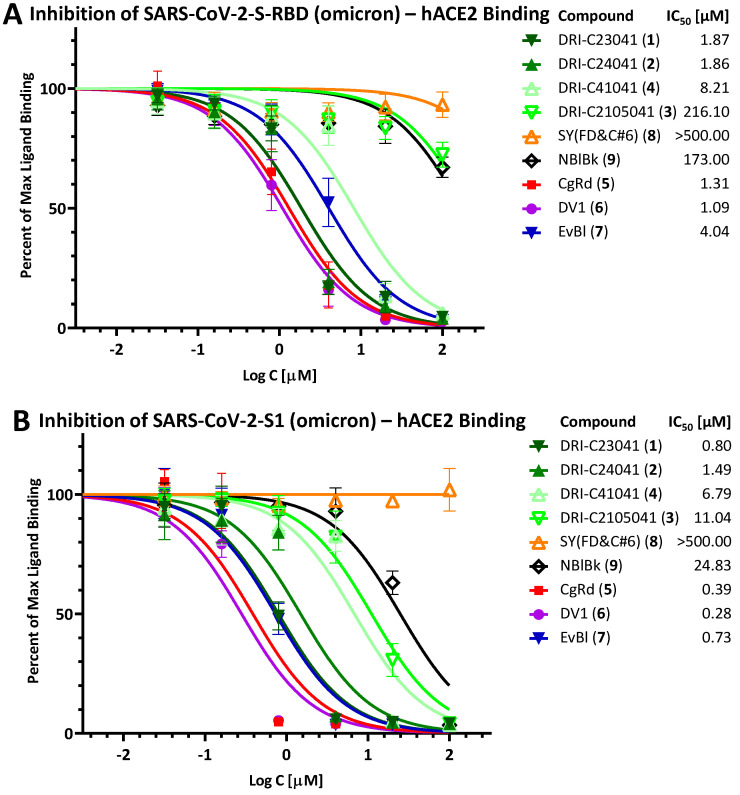
**Inhibition of SARS-CoV-2-S binding (omicron, B.1.1.529) to hACE2.** Concentration-response curves obtained for the inhibition of the PPI between SARS-CoV-2-S-RBD (omicron) (**A**) or SARS-CoV-2-S1 (omicron) (**B**) and hACE2 as done for the original strain in Figure 2.

Due to the increased importance of broad-spectrum activity, especially following the emergence of VoCs for SARS-CoV-2, we also tested these compounds to assess their inhibitory activity against the corresponding PPIs with mutant spike proteins including D614G, delta (B.1.617.2), and omicron (B.1.1.529). Just as for the original strain, we assessed activity using both the RBD and the S1 fragments (except for D614G, where this was not possible as the mutation is only in the S1 and not in the RBD region). Notably, both DRI-C23041 (**1**) and DRI-C24041 (**2**) maintained their low micromolar activity in all of these assays (IC_50_ < 3.0 μM), as shown in Figure 3 (D614G), Figure 4 (delta), and Figure 5 (omicron), indicating their potential for broad-spectrum activity. Similarly, the organic dyes CgRd, DV1, and EvBl (**5**–**7**), which showed good activity against the original strain (Figure 2B), retained their low-micromolar activity for these VoC PPIs as well (Figure 3, Figure 4 and Figure 5). The negative control SY(FD&C#6) (**8**) remained consistently inactive in all assays (IC_50_ > 500 μM). In the meantime, just as for the original strain, DRI-C41041 (**4**), DRI-C2105041 (**3**), and NBlBk (**9**) showed little if any activity in the assays with RBDs, but some (mid-micromolar) activity in the S1 assays, indicating again somewhat more susceptibility to inhibition for the S1 fragment as compared to RBD only.

### 2.3. Inhibition of HCoV-NL63 S—hACE2 PPI

**Figure 6:**
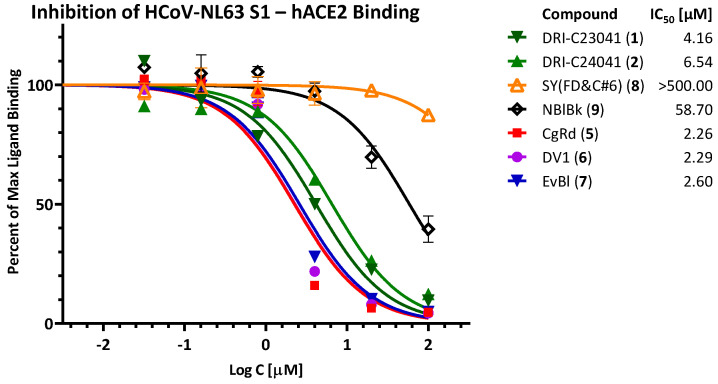
**Inhibition of HCoV-NL63 S binding to hACE2.** Concentration-response curves obtained for the inhibition of the PPI between HCoV-NL63 S1 and hACE2 as done for SARS-CoV-2 in previous figures.

Finally, since there is one other known coronavirus that infects humans, causing a common cold that also uses hACE2 as its receptor for attachment and entry (HCoV-NL63), we also assessed the inhibitory activity of some of these compounds against the HCoV-NL63 S1—hACE2 PPI (Figure 6). As the affinity of the HCoV-NL63 S1 to hACE2 is considerable less than that of SARS-CoV-2 S1 (46 vs. 16 nM in our setup [36]), larger protein amounts were needed for detectability; therefore, we assessed only a subset of compounds of interest. Notably, DRI-C23041 and DRI-C24041 lost some (2–5-fold) activity compared to the SARS-CoV assays, but still showed good inhibition (IC_50_ values of 4.16 and 6.54 μM, respectively), indicating possible pan-coronavirus inhibitory activity. The organic dyes CgRd, DV1, and EvBl, which we have shown before to be promiscuous and non-specific PPI inhibitors [36], maintained their low micromolar inhibitory activity (IC_50_ < 3.0 μM).

### 2.4. Inhibition of SARS-CoV-2 Pseudovirus Entry (Original Strain and Delta VoC)

**Figure 7:**
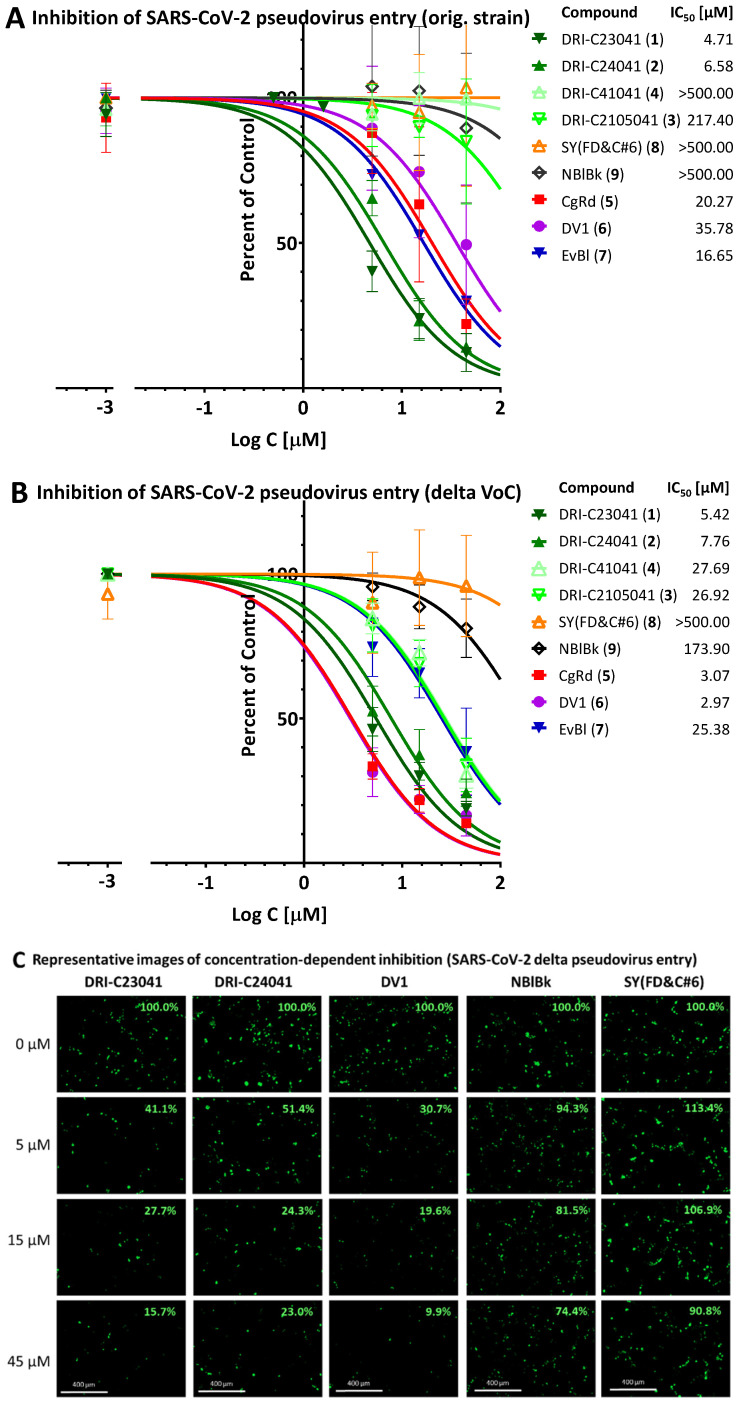
**Concentration–dependent inhibition of SARS-CoV-2 pseudovirus entry into hACE2-expressing cells for the original strain (A) and the delta VoC (B,C).** Quantification of entry of pseudoviruses bearing the SARS-CoV-2 S protein (plus green fluorescent protein reporters) in hACE2-expressing host cells (HEK293T). Concentration-response curves for the original strain and the delta VoC are shown in (**A**,**B**), respectively; they were obtained by fitting the quantification of pseudovirus entry (green) as estimated using ImageJ (mean ± SD from three independent experiments). Because green fluorescence is expressed only in cells infected by the pseudovirus, the amount of green present is proportional with the number of infected cells. Illustrative images from different individual experiments are shown for DRI-C23041, DRI-C24041, DV1, NBlBk, and SY (FD&C#6) in (**C**) (numbers within each panel indicating percent compared to corresponding controls in top row as 100%).

Next, we confirmed inhibitory activities using a cell-based pseudovirus assay that allows the quantification of viral entry without having to use biosafety level 3 (BSL-3) or higher containment since it uses pseudoviruses that do not replicate in human cells. As before [36], this has been done with a baculovirus pseudotyped with SARS-CoV-2-S proteins and generated using BacMam-based tools. If the nuclei of pseudovirus-exposed host cells (here, ACE2- and red fluorescence expressing HEK293T) show green fluorescence, pseudovirus entry was completed. If entry is blocked, the cell nuclei remain dark. Here, we tested inhibitory activities using pseudoviruses expressing the SARS-CoV-2 spike protein corresponding to the original strain and the delta variant (Figure 7). 

In agreement with their good PPI inhibitory activity, DRI-C23041 (**1**) and DRI-C24041 (**2**) showed good concentration-dependent inhibitory activity with similar IC_50_ values for both the original strain (4.71 μM and 6.58 μM, respectively) and the delta (B.1.617.2) variant (5.42 μM and 7.76 μM, respectively). CgRd (**5**), DV1 (**6**), and EvBl (7) also inhibited, the first two showing considerably better activity for the delta variant (3.07 μM and 2.97 μM, respectively) than for the original strain (20.27 μM and 35.78 μM, respectively). Following the same trend, while DRI-C41041 and DRI-C2105041 showed very little inhibition for the original strain (IC_50_ > 500 μM and 217.4 μM), they had some activity against the delta variant (27.69 μM and 26.92 μM, respectively) (Figure 7). The negative control SY(FD&C#6) did not show any significant inhibition either for the original strain or for the delta variant (IC_50_ > 500 μM). NBlBk also had essentially no inhibitory activity, showing only some very limited inhibition for the delta variant (IC_50_ > 500 μM and 173.9 μM, respectively).

**Figure 8:**
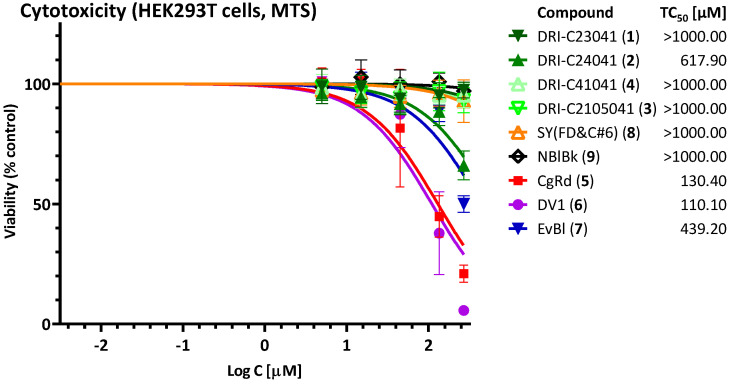
**Cytotoxicity assessment.** MTS cytotoxicity assay (HEK293T cells) using the same conditions as for the activity assessment (Figure 7). Data (symbols) are average ± SD of three experiments in duplicates; TC_50_ values shown in the legend were obtained from fitting with standard sigmoid curves as before.

**Figure 9:**
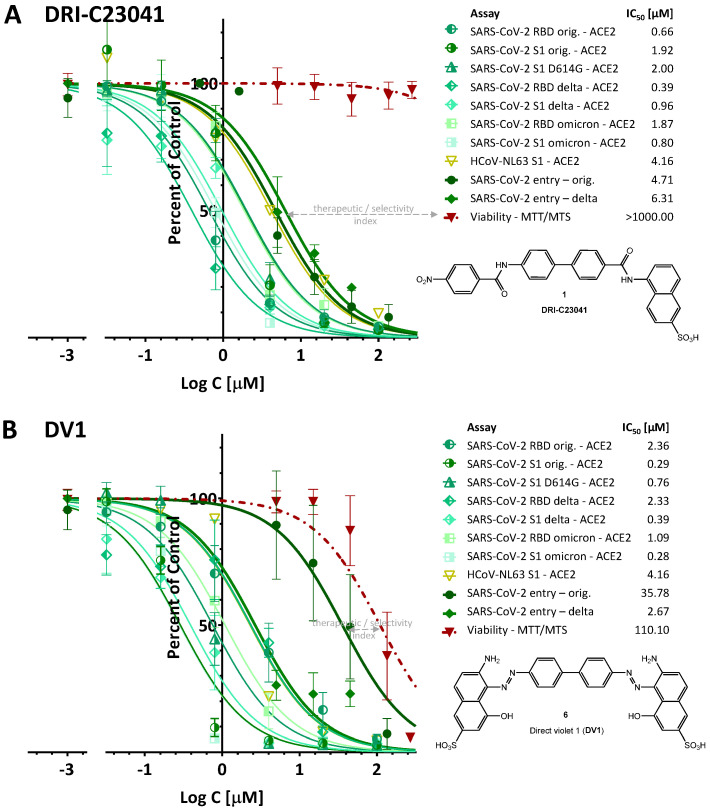
**Overall PPIs and viral entry inhibitory activity of DRI-C23041 (A) with DV1 included for comparison (B).** Concentration-dependent inhibition of the spike–hACE2 PPIs assessed here (thinner lines) shown in parallel with the cell-based pseudovirus entry inhibitions (thicker dark green lines) and cytotoxicity (dark red) to illustrate the therapeutic (selectivity) index. Data are the same as in the previous figures just collected here in the same graph to allow for clear comparison and highlighting of the therapeutic index for each compound.

In parallel with this, we also evaluated the cytotoxicity of these compounds under conditions similar to those used to evaluate their activity to assess their therapeutic (selectivity) index, TI (SI) = TC_50_/IC_50_. None of the DRI-C compounds tested showed significant effect on the viability of HEK293T cells, with DRI-C24041 being the most toxic (TC_50_ > 600 μM) and all others having TC_50_ > 1000 μM (Figure 8). Accordingly, DRI-C23041 shows particularly promising separation between activity and toxicity, i.e., therapeutic index, as illustrated in Figure 9A, which summarizes all its activity in a single graph. In agreement with their promiscuous PPI inhibitory activity, the organic dyes CgRd, DV1, and EvBl showed more pronounced cytotoxicity in the HEK293T cells as tested here (TC_50_: 130, 110, and 439 μM, respectively). Accordingly, there is much less separation between their inhibitory and toxic activities, as illustrated for DV1 in Figure 9B.

## 3. Discussion

Our results here reconfirmed that the DRI-C compounds we identified earlier, such as DRI-C23041 (**1**) as well as some organic dyes such as CgRd (**5**) and DV1 (**6**), have potential as SMIs of PPIs mediating the cell attachment and entry of SARS-CoV-2 and possibly even other coronaviruses. DRI-C23041 in particular showed promising separation between activity and non-specific cytotoxicity, having TI >> 100 (Figure 9A), but even DRI-C24041 has TI ≈ 100 as calculated based on the ratio of the TC_50_ and IC_50_ values. The organic dyes, especially CgRd and DV1, also show good inhibitory activity; however, they seem to act as non-specific and promiscuous PPI inhibitors. Accordingly, they also have a quite narrow therapeutic index and, thus, low therapeutic promise (Figure 9B). Nevertheless, this work confirms again that, as highlighted before, by being rich in privileged structures for protein binding, the chemical space of organic dyes provides a useful starting point in the search for effective SMI scaffolds for PPI inhibition in general [15,36,41]—more so than typical drug-like screening libraries. This is also corroborated by comparing these results obtained by us from screening a relatively limited number of compounds (<200) with those obtained by other efforts to identify SMIs of spike—hACE2 PPIs using various screening approaches that often involved multiple thousands of candidate compounds, which have been reviewed recently [15]. So far, SMIs that have antiviral activity with an IC_50_ < 30 µM confirmed in a live viral or pseudoviral assay are limited to DRI-C23041 (**1**) [36] and some organic dyes, including Congo red (**5**), direct violet 1 (**6**), Evans blue (**7**), and methylene blue, from our work [36,37,38]; methylene blue [55,56,57,58] and Evans blue [59] also found by others; verteporfin [60]; and cannabigerolic and cannabidiolic acids [61]. It is notable, for example, that Evans blue was established as the best hit identified from a high-throughput screening of more than 3000 compounds, which were themselves preselected after in silico prescreening of ~60,000 structures [59], whereas it ranked as not even the most promising hit in our screening of a much smaller library of slightly more than a hundred organic dyes. Results obtained there (*K*_D_ = 2.2 μM for binding to SARS-CoV-2-S and IC_50_ = 28.1 μM for SARS-CoV-2 infection inhibition in Vero E6 cells [59]) are quite consistent with those obtained here (Figure 2 and Figure 7). Finally, regarding organic dyes as SMIs of PPIs, it is also of interest that methylene blue was found to inhibit the entry and replication of SARS-CoV-2, and has potential as a possible inexpensive, broad-spectrum antiviral for the prevention and treatment of COVID-19, possibly due to multiple mechanisms of action [37,38,55,56,57,58], even if it is a promiscuous PPI inhibitor with a fairly narrow TI [38], since it is FDA approved for clinical use and is included in the *WHO List of Essential Medicines*.

Of course, structures identified from organic dye libraries will need considerable further structural optimization to eliminate their color, increase their specificity, and optimize their clinical potential [34,35]. SMIs of PPIs tend to be larger and more hydrophobic structures than what is considered typical for drug-like molecules, because such larger structures that typically contain multiple aromatic rings are usually needed for efficient protein binding and PPI inhibition [52,53,54]. Thus, they also tend to violate the so-called “rule-of-five” (Ro5) criteria widely applied to ensure adequate oral bioavailability and pharmacokinetic profile during lead candidate selection, since Ro5 criteria include, among others, the need to have a molecular weight less than 500 Daltons [62,63]. Nevertheless, several new drugs have been launched in the last decade or so that violate these self-imposed empirical rules and prove that oral bioavailability can be achieved in what is called the “beyond the Ro5” chemical space, even if not easily [64,65]. Thus, when looking at the potential of development and clinical translatability of identified PPI SMI hits, it is worth noting that it will most likely involve considerable optimization work anyway. For example, ABT-737, which was the first lead compound during the development process of venetoclax (ABT-199), was so far from suitable for formulation as a typical drug that it was described as having “the biophysical properties of brick dust” [66]. Similarly, it took an impressively tedious medicinal chemistry optimization process to make the original lead of the series into what ultimately became the clinical product fostemsavir (BMS-663068), as neatly summarized in [44]. As a first evaluation of the drug-likeness and clinical translatability of the present compounds, summaries of their physicochemical and absorption, distribution, metabolism, and excretion plus toxicity (ADMET) properties calculated by SwissADME [67] and ADMETlab2.0 [68] (accessed on 19 August 2022) are included in Supplementary Information Table S1.

Finally, it should be mentioned that results here indicate that it seems feasible that SMI structures can be identified that have sufficiently broad-spectrum activity, i.e., they can inhibit several variants and mutants of SARS-CoV-2. In fact, it could be possible to find SMI that can also inhibit other coronaviruses, including not just SARS-CoV and MERS-CoV but even less dangerous ones such as the common cold-causing HCoVs (e.g., Figure 6). Ultimately, it is hoped that such inhibitory effects on coronaviral attachment can be translated into antiviral activity against the COVID-19-causing SARS-CoV-2 and maybe even other CoVs that bind to ACE2 such as SARS-CoV and the α-coronavirus HCoV-NL63. 

## 4. Materials and Methods

### 4.1. Binding Assays

The DRI-C compounds used here were synthesized as described before [36]. Organic dyes were purchased from the manufacturer as listed below with their purities and catalog numbers in parenthesis: Congo red (85%, 860,956), naphthol blue black (>99%, 70,490), sunset yellow FCF (90%, 465,224), and Evans blue (85%, 206,334) were from Sigma-Aldrich (St. Louis, MO, USA); direct violet 1 (>99%, C0551) was from TCI America (Portland, OR, USA). All CoV proteins used were purchased from Sino Biological (Wayne, PA, USA) and are as follows: human ACE2-Fc (Cat. no. 10108-H05H), SARS-CoV-2 Spike S1 (His tag, Cat. no. 40591-V08H), RBD (His tag, Cat. no. 40592-V08H), D614G S1 (His tag, Cat. no. 40591-V08H3), delta S1 (His tag, Cat. no. 40591-V08H23), delta RBD (His tag, Cat. no. 40592-V08H90), omicron S1 (His tag, Cat. no. 40591-V08H41), omicron RBD (His tag, Cat. no. 40592-V08H121), and HCoV-NL63 S1 (His tag, Cat. no. 40600-V08H). The mutation sites of the delta variant are RBD: L452R and T478K; S1: T19R, G142D, E156G, 157–158 deletion, L452R, T478K, D614G, and P681R. The mutation sites of the omicron variant are RBD: G339D, S371L, S373P, S375F, K417N, N440K, G446S, S477N, T478K, E484A, Q493R, G496S, Q498R, N501Y, and Y505H; S1: A67V, HV69-70 deletion, T95I, G142D, VYY143-145 deletion, N211 deletion, L212I, ins214EPE, G339D, S371L, S373P, S375F, K417N, N440K, G446S, S477N, T478K, E484A, Q493R, G496S, Q498R, N501Y, Y505H, T547K, D614G, H655Y, N679K, and P681H.

The binding inhibition assays were performed as described before [33,34,36,38]. Briefly, 96-well microtiter plates (Nunc F Maxisorp; Thermo Fisher Scientific, Waltham, MA, USA) were precoated with Fc-conjugated ACE2 receptor (100 μL/well) and kept at 4 °C overnight. They were washed once with washing buffer (PBS pH 7.4, 0.05% Tween-20) and then blocked with 2% BSA (A7030, Sigma-Aldrich, St. Louis, MO, USA) for 1 h at room temperature. Plates were then washed twice with washing buffer and mixed corresponding concentration of His-tagged ligands with test compounds diluted in binding buffer (20 mM HEPES, pH 6.8) to give a final volume of 100 μL/well and incubated for 1 h. After incubation, the plates were washed three times and incubated with anti-His HRP conjugate (1:20,000 dilution) (Cat. no. 652504; BioLegend; San Diego, CA, USA) for 1 h. Plates were then washed four times, added with HRP substrate TMB (3,3′,5,5′-tetramethylbenzidine), and protected from light for up to 15 min. Finally, the reaction was terminated by adding 1 M H_2_SO_4_ and the absorbance value was read at 450 nm. Fc-conjugated receptor protein concentrations used for inhibition assay were 1.0 μg/mL ACE2 for SARS-CoV-2 Spike RBD, D614G S1 variant, and omicron S1 variant; 0.5 μg/mL ACE2 for delta Spike RBD and S1, and omicron Spike RBD; and 2.0 μg/mL ACE2 for HCoV-NL63. The concentrations of protein ligands used here were 0.5 μg/mL for original SARS-CoV-2 Spike RBD, delta RBD, and omicron S1; 1.0 μg/mL for D614G S1 variant, delta S1, and omicron RBD; and 20 μg/mL for HCoV-NL63. These concentrations were chosen from preliminary experiments optimizing the response (i.e., to achieve adequate signal at conditions close to the EC_50_). All compounds were prepared in dimethyl sulfoxide (DMSO) at 10 mM stock solution. 

### 4.2. SARS-CoV-2 Pseudovirus Assays

To perform the pseudovirus experiments, fluorescent biosensors for ACE2 (Cat. no. C1100R), pseudo SARS-CoV-2 green reporter (Cat. no. C1110G), and pseudo SARS-CoV-2 spike delta variant green reporter (Cat. no. C1123G) were used, all from Montana Molecular, Bozeman, MT, USA. They were applied according to the manufacturer’s instruction with minor modifications as described before [36,37]. Mutation sites of the delta variant tested here are T19R, V70F, T95I, G142D, E156-, F157-, R158G, A222V, W258L, K417N, L452R, T478K, D614G, P681R, and D950N.

### 4.3. Cytotoxicity Assay

HEK293T cells were seeded onto 96-wells and treated with five concentrations of test compounds (5, 15, 45, 135, and 270 µM) for 48 h. Old media were removed and replaced with fresh ones for additional 48 h. Then, cells were treated with MTT solution at 37 °C for 2 h. Cell viability was determined by optical density at 570 nm using a microplate reader (SpectraMax iD3, Molecular Devices, San Jose, California, CA, USA).

### 4.4. Statistical Analysis and Data Fitting

All binding and pseudovirus entry inhibition assays were performed as duplicates or triplicates per plate, and all experiments were repeated at least three independent times (with the exception of the HCoV-NL63 S1—hACE2 assay, where, due to the larger protein amounts needed, only two repeats were done and only for a subset of compounds). As before [34,36,38], data were normalized as inhibition % and fitted with standard concentration-response models in GraphPad Prism (GraphPad, La Jolla, CA, USA) to determine EC_50_ or IC_50_ values (half-maximal effective or inhibitory concentrations).

## 5. Conclusions

In conclusion, even if specificities and activities of the compounds identified here still require further optimization, these results provide proof-of-principle evidence for the feasibility of SMIs targeting the coronavirus spike protein—hACE2 PPI that could lead to alternative, orally bioavailable therapies for the prevention and treatment of COVID-19 and maybe even other diseases caused by coronavirus infection.

## 6. Patents

Buchwald, P. Small molecule inhibitors of coronavirus attachment and entry, methods and uses thereof. International Patent Application, International Patent Application No. PCT/US21/52520, Filed 29 September 2021.

## Data Availability

Data is contained within the article and supplementary material.

## Supplementary Materials

The following supporting information can be downloaded at: https://www.mdpi.com/article/10.3390/ph15091084/s1, Table S1: Physicochemical and ADMET properties calculated for the present compounds (**1**–**9**) by SwissADME and ADMETlab2.0 (accessed on 19 August 2022).

## Abbreviations

The following abbreviations are used in this manuscript:

ACE2	angiotensin converting enzyme 2
CgRd	Congo red
CoV	coronavirus
DV1	direct violet 1
EvBl	Evans blue
NBlBk	naphthol blue black
PPI	protein–protein interaction
RBD	receptor binding domain
SARS	severe acute respiratory syndrome
SMI	small-molecule inhibitor
SY	sunset yellow FCF
VoC	variant of concern
WHO	World Health Organization
